# No effect of ascorbate on cutaneous vasodilation and sweating in older men and those with type 2 diabetes exercising in the heat

**DOI:** 10.14814/phy2.13238

**Published:** 2017-04-10

**Authors:** Naoto Fujii, Robert D. Meade, Pegah Akbari, Jeffrey C. Louie, Lacy M. Alexander, Pierre Boulay, Ronald J. Sigal, Glen P. Kenny

**Affiliations:** ^1^ Human and Environmental Physiology Research UnitSchool of Human KineticsUniversity of OttawaOttawaOntarioCanada; ^2^ Institute of Health and Sport SciencesUniversity of TsukubaTsukuba CityJapan; ^3^ Department of KinesiologyNoll LaboratoryPennsylvania State UniversityUniversity ParkPennsylvania; ^4^ Faculty of Physical Activity SciencesUniversity of SherbrookeSherbrookeCanada; ^5^ Departments of MedicineCardiac Sciences and Community Health SciencesFaculties of Medicine and KinesiologyUniversity of CalgaryCalgaryAlbertaCanada; ^6^ Clinical Epidemiology ProgramOttawa Hospital Research InstituteOttawaOntarioCanada; ^7^ Present address: Institute of Health and Sport SciencesUniversity of TsukubaTsukuba CityJapan

**Keywords:** Aging, heat loss, microcirculation, reactive oxygen species, sweating, thermoregulation

## Abstract

Aging and chronic disease such as type 2 diabetes (T2D) are associated with impairments in the body's ability to dissipate heat. To reduce the risk of heat‐related injuries in these heat vulnerable individuals, it is necessary to identify interventions that can attenuate this impairment. We evaluated the hypothesis that intradermal administration of ascorbate improves cutaneous vasodilation and sweating in older adults via nitric oxide synthase (NOS)‐dependent mechanisms during exercise in the heat and whether these improvements, if any, are greater in individuals with T2D. Older males with (*n* = 12, 61 ± 9 years) and without (*n* = 12, 64 ± 7 years) T2D performed two 30‐min bouts of cycling at a fixed rate of metabolic heat production of 500 W (~70% peak oxygen uptake) in the heat (35°C); each followed by a 20‐ and 40‐min recovery, respectively. Cutaneous vascular conductance (CVC) and sweat rate were measured at four intradermal microdialysis sites treated with either (1) lactated Ringer (Control), (2) 10 mmol/L ascorbate (an antioxidant), (3) 10 mmol/L _L_‐NAME (non‐selective NOS inhibitor), or (4) a combination of ascorbate + _L_‐NAME. In both groups, ascorbate did not modulate CVC or sweating during exercise relative to Control (all *P* > 0.05). In comparison to Control, _L_‐NAME alone or combined with ascorbate attenuated CVC during exercise (all *P* ≤ 0.05) but had no influence on sweating (all *P* > 0.05). We show that in both healthy and T2D older adults, intradermal administration of ascorbate does not improve cutaneous vasodilation and sweating during exercise in the heat. However, NOS plays an important role in mediating cutaneous vasodilation.

## Introduction

Older adults are at greater risk of heat‐related injuries (e.g., heat syncope and heat stroke), likely due to attenuated cardiovascular and thermoregulatory function relative to their younger counterparts (Worfolk 2000; Kenney et al. 2014; Kenny et al. 2016a). In parallel, although nitric oxide (NO) synthase (NOS) is known to contribute to the regulation of cutaneous vasodilation (Kellogg et al. 1998; Shastry et al. 2000; Welch et al. 2009; Fujii et al. 2014b; McNamara et al. 2014) and sweating (Welch et al. 2009; Fujii et al. 2014b; Stapleton et al. 2014; Louie et al. 2016) during heat stress in young adults, these contributions are diminished in older adults (Stanhewicz et al. 2013; Stapleton et al. 2014; Fujii et al. 2015b, 2016b). As a potential mechanism, aging is known to increase the level of reactive oxygen species (i.e., oxidative stress) such as superoxide (Durrant et al. 2009), which can reduce NO bioavailability (Fujii et al. 2014a). Hence, in older adults, the nonselective antioxidant ascorbate may improve cutaneous vasodilation and sweating via NOS‐dependent mechanisms during heat stress, such as that which occurs during exercise in the heat.

Individuals with type 2 diabetes mellitus are at increased risk of heat‐related injuries (Semenza et al. 1999; Kenny et al. 2016b). This is likely secondary to an attenuation in whole‐body heat dissipation (Kenny et al. 2013) associated with reductions in local cutaneous vasodilation (Wick et al. 2006; Sokolnicki et al. 2009) and sweating (Fealey et al. 1989; Petrofsky et al. 2005); albeit due to regional heterogeneity, reductions in local heat loss responses are not always observed (Kenny et al. 2013, 2016b). Individuals with type 2 diabetes have been shown to have increased oxidative stress relative to healthy older adults without type 2 diabetes (Folli et al. 2011; Casoinic et al. 2016; Restaino et al. 2016), which as mentioned above, may result in attenuations in the heat loss responses via mechanisms related to NO. Ascorbate may therefore augment the heat loss responses during exercise in the heat in older adults with type 2 diabetes to a greater extent compared to healthy older adults.

This study evaluated the effect of local ascorbate administration on the regulation of cutaneous vasodilation and sweating response during exercise in the heat in older adults with and without type 2 diabetes. We hypothesized that reducing oxidative stress improves the heat loss responses of cutaneous vasodilation and sweating by increasing NOS‐derived NO in both healthy older adults and those with type 2 diabetes during exercise in the heat, with the latter group experiencing comparatively greater improvements.

## Materials and Methods

### Ethical approval

This study was approved by the University of Ottawa Health Sciences and Science Research Ethics Board and adhered to the guidelines set forth by the *Declaration of Helsinki*. Verbal and written informed consent was acquired from all volunteers before the initiation of any experimental procedures.

### Participants

Twelve older males diagnosed with type 2 diabetes mellitus (T2D) for at least 5 years and twelve healthy nondiabetic older males (Control) participated in this study. All participants were free of respiratory disease, heart disease, uncontrolled hypertension, and neuropathy. Participant characteristics are described in Table 1. All participants with T2D were taking the following medications (number of subjects are indicated in parenthesis): metformin (12), statin (6), dipeptidyl peptidase‐4 inhibitor (6), sulfonylurea (6), angiotensin converting enzyme inhibitor (4), acetylsalicylic acid (4), insulin (3), angiotensin receptor blocker (3), calcium channel blocker (2), sodium/glucose cotransporter 2 inhibitor (1), beta‐blocker (1), and diuretic (1). All subjects in the Control group were without prescription medications. All participants were nonsmoking for at least 5 years.

**Table 1:** Participant characteristics

	Control group	T2D group
Number of participants	12	12
Age (years)	64 ± 7	61 ± 9
Height (m)	1.75 ± 0.05	1.70 ± 0.06^a^
Body mass (kg)	83.0 ± 8.5	87.5 ± 18.7
Body surface area (m^2^)	1.99 ± 0.11	1.98 ± 0.22
Body fat (%)	31 ± 6	30 ± 6
Peak oxygen uptake (L/min)	2.6 ± 0.3	2.3 ± 0.5
(mL/kg/min)	31.9 ± 4.2	27.0 ± 4.8^a^
HbA_1C_ (%)	–	7.4 ± 1.1
Duration of diabetes (years)	n/a	7.5 ± 4.4

All values are expressed as mean ± standard deviation. T2D, type 2 diabetes. HbA_1C_, hemoglobin A_1c_. n/a, not applicable.

a versus Control group (*P* ≤ 0.05).

### Experimental design

All participants completed one preliminary and one experimental session on separate days. Participants were asked to refrain from consuming alcohol, caffeine, and heavy exercise at least 12 h prior to the start of the sessions. On the day of each session, participants were allowed to have food 2 h before the experiment. Experimental trials were performed at different periods during the day depending on individual schedules. During the preliminary session, body mass, height, body surface area, body fat, and peak oxygen uptake were determined. Body height was measured using a stadiometer (Model 2391, Detecto Scale Company, Webb City, MO), and body mass was measured by a digital weight scale platform (model CBU150X, Mettler Toledo Inc., Mississauga, ON, Canada). Body surface area was calculated using body mass and height (DuBois and DuBois 1916). Hydrostatic weighing was used to estimate body fat percentage (Siri 1956). Peak oxygen uptake on a semirecumbent cycle ergometer (Corival Recumbent, Lode B.V., Groningen, Netherlands) was evaluated using an automated indirect calorimetry system (Medgraphics Ultima, Medical Graphics Corporation, St Paul, MN) as previously reported (Fujii et al. 2016a).

On the day of the experimental session, body mass was measured then participants changed into shorts and running shoes. Thereafter, participants were transferred to a thermoneutral room (~23°C) where they were seated in a semirecumbent position and were instrumented with four microdialysis fibers (MD2000, Bioanalytical Systems, West Lafayette, IN) in the dermal layer of the skin on the dorsal side of the left forearm. These fibers have a semipermeable membrane (30 kDa cutoff, 10 mm), through which pharmacological agents can be continuously delivered into the dermis, ultimately affecting skin vessels and sweat glands. In order to situate the fibers subcutaneously, a 25‐gauge needle was inserted into the skin using aseptic technique. The entry and exit points of fiber insertion were separated by ~2.5 cm. The microdialysis fiber was then threaded through the lumen of the needle, after which the needle was withdrawn leaving the fiber in place. The location of each microdialysis fiber was separated by 2–4 cm.

Once all four fibers were placed in the skin, each microdialysis site was perfused with lactated Ringer (Baxter, Deerfield, IL) at a constant rate of 4 *μ*L/min with a micro‐infusion pump (Model 400, CMA Microdialysis, Solna, Sweden) for approximately 30 min. Thereafter, the participants moved to a thermal chamber (Can‐Trol Environmental Systems Limited, Markham, ON, Canada) regulated to an ambient air temperature of 35°C and a relative humidity of 20% where they rested on a semirecumbent cycle ergometer (Corival Recumbent, Lode B.V.) and perfusion of pharmacological agents through each microdialysis fiber began. The four sites were continuously perfused in a counterbalanced manner with either (1) lactated Ringer (Baxter, Deerfield, IL, US) (vehicle control), (2) 10 mmol/L ascorbate, a known antioxidant (Sigma‐Aldrich, St. Louis, MO), (3) 10 mmol/L *N* ^G^‐nitro‐_L_‐arginine methyl ester (_L_‐NAME), a nonselective NOS inhibitor (Sigma‐Aldrich), or (4) a combination of 10 mmol/L ascorbate and 10 mmol/L _L_‐NAME (ascorbate + _L_‐NAME). These concentrations were determined based on previous studies in which intradermal microdialysis was employed in human skin (Holowatz and Kenney 2007; Stewart et al. 2008; Yamazaki 2010; Medow et al. 2011; Fujii et al. 2015a; Meade et al. 2015). Each infusion was performed at a constant rate of 4.0 *μ*L/min for at least 60 min prior to data collection to ensure action of each drug. Furthermore, ~90 min elapsed between fiber insertion and the beginning of baseline data collection (30 min of lactated Ringer infusion at all sites + 60 min drug perfusion), a period of time sufficient to ensure that any tissue redness associated with fiber insertion had subsided (Anderson et al. 1994). Since this drug infusion was performed in the heat (35°C), the participants were pre‐exposed to a sufficiently prolonged ambient heat stress prior to data collection. As such, we were also able to assess the influence of a passive heat stress on heat loss responses which has been shown to be attenuated in older adults with (Kenny et al. 2016a) and without type 2 diabetes (Kenny et al. 2015) relative to young adults.

After the >60 min drug infusion period, 10‐min of baseline resting measurements were taken in the heat (35°C). Thereafter, the participants completed two 30‐min bouts of cycling at a fixed rate of metabolic heat production of ~500 W (Control: 480 ± 32; T2D: 500 ± 7 W; *P* = 0.12). A fixed heat load was employed to ensure a similar thermal drive, and therefore stimulus for sweating, between individuals (Gagnon et al. 2013). This resulted in a similar exercise intensities as defined by %peak oxygen uptake in the Control and T2D groups of 68 ± 4 and 72 ± 5%, respectively (*P* = 0.22). The first and second bouts of exercise were followed by 20‐ and 40‐min recovery periods. In this study we employed two bouts of exercise in order to evaluate the mechanisms underpinning the regulation of heat loss responses during and following successive exercise cycles. Studies show that the heat loss responses are rapidly attenuated in the first 15‐min following cessation of exercise despite progressive increases in body core temperature with successive exercise bouts. However, the heat loss responses are activated more quickly in subsequent exercise bouts. These differences have been shown to have an important effect on body core temperature regulation. We employed a longer 40‐min recovery period following the second exercise bout to ensure that stable values had been achieved. Following the second 40‐min recovery period, 50 mmol/L sodium nitroprusside (Sigma‐Aldrich) was administered at all four microdialysis sites at a rate of 6.0 *μ*L/min for 20–30 min in order to achieve maximal cutaneous vasodilation. Thereafter, all equipment were removed, and post‐experiment body mass was recorded.

### Measurements

We employed the 1.1 cm^2^ ventilated sweat capsule which was designed for the evaluation of local forearm sweat rate when employing intradermal microdialysis (Meade et al. 2016). Local sweat rate was estimated based on sweat production evaluated under the sweat capsules, which were positioned over the center of each microdialysis membrane and affixed to the skin with adhesive rings and topical skin glue (Collodion HV, Mavidon Medical products, Lake Worth, FL). To evaporate all sweat produced under the capsule, dry compressed air from gas tanks located in the thermal chamber was supplied to each capsule at a constant rate. The water content of the effluent air was measured with a capacitance hygrometer (Model HMT333, Vaisala, Helsinki, Finland). Local forearm sweat rate was calculated every 5 sec using the difference in humidity between influent and effluent air, multiplied by the flow rate, and normalized to the skin surface area under the capsule (mg/min/cm^2^).

Cutaneous red blood cell flux, expressed in perfusion units (index of cutaneous blood flow), was locally measured at a sampling rate of 32 Hz with laser‐Doppler flowmetry (PeriFlux System 5000, Perimed, Stockholm, Sweden). An integrated laser‐Doppler flowmetry probe with a 7‐laser array (Model 413, Perimed) was placed in the center of each sweat capsule directly over each microdialysis fiber. Systolic and diastolic blood pressures were measured by manual auscultation using a mercury column sphygmomanometer (Baumanometer Standby Model, WA Baum Co, Copiague, NY). Mean arterial pressure was calculated as diastolic arterial pressure plus one‐third the difference between systolic and diastolic pressures (pulse pressure). Cutaneous vascular conductance (CVC) was calculated as cutaneous red blood cell flux divided by mean arterial pressure. CVC data were normalized at each site as percentage of maximum (expressed as %CVCmax). Maximum CVC was obtained during the administration of sodium nitroprusside at the end of the experiment. Heart rate was recorded every 5 sec using a heart rate monitor (RS400, Polar Electro, Kempele, Finland).

Body core temperature was measured using a thermocouple temperature probe (Mon‐a‐therm, Mallinckrodt Medical, St Louis, MO) inserted in the esophagus or rectum. For participants unable to tolerate the insertion of these probes, a temperature sensor (Braun ThermoScan PRO 6000, Welch Allyn, Skaneateles Falls, New York) inserted in the aural canal was employed to estimate body core temperature. Skin temperature was measured using thermocouples (Concept Engineering, Old Saybrook, CT) attached to the skin with adhesive rings and surgical tape. Mean skin temperature was estimated as a weighted mean value using local skin temperatures measured at four sites (20% calf, 20% quadriceps, 30% biceps, and 30% chest) according to the equation used by Hardy and Dubois (1938). Esophageal, rectal, and skin temperature data were sampled at 15 sec intervals using a data acquisition module (Model 34970A; Agilent Technologies Canada Inc., Mississauga, ON, Canada) and simultaneously displayed and recorded in spreadsheet format on a personal computer with LabVIEW software (Version 7.0, National Instruments, Austin, TX). Aural canal temperature was recorded every 5 min.

Metabolic rate was determined using indirect calorimetry (MOXUS system, Applied Electrochemistry, Pittsburgh, PA) where expired gases were analyzed for oxygen and carbon dioxide concentrations. Approximately 20 min before the start of baseline resting data collection, gas mixtures of known concentrations were used to calibrate gas analyzers and a 3 L syringe was used to calibrate the turbine ventilometer. The subjects wore full face masks (Model 7600 V2, Hans‐Rudolph, Kansas City, MO) attached to a 2‐way T‐shape nonrebreathing valve (Model 2700, Hans‐Rudolph). Oxygen uptake and respiratory exchange ratio were obtained every 30 sec and were used to calculate metabolic rate (Nishi 1981; Kenny and Jay 2013). Metabolic heat load was estimated as metabolic rate minus the external work (i.e., work rate during cycling).

For the safety of participants with type 2 diabetes, blood glucose concentration was monitored using a blood glucose meter (Contour Next, Bayer AG, Leverkusen, Germany).

### Data analysis

All data used for data analysis were obtained by averaging values over the last 5 min of each time period, with the exception of absolute maximal CVC (perfusion units^**.**^mmHg^−1^) obtained during sodium nitroprusside administration (peak values averaged over 2 min). The contribution of NOS to cutaneous vasodilation occurring during the last 5 min of each exercise bout was evaluated as the difference (∆) in CVC between the vehicle control and _L_‐NAME sites. Similarly, the NOS contribution in the presence of ascorbate was also evaluated by ∆CVC between the vehicle control and ascorbate + _L_‐NAME sites. Due to technical difficulties, the measurement of one CVC, heart rate, body core temperature, and percent body fat as well as three skin temperatures from the T2D group, and one body core temperature from the Control group were not acquired.

### Statistical analysis

Local forearm CVC and sweat rate were analyzed using a three‐way mixed‐design analysis of variance with factors of treatment site (four levels: vehicle control, ascorbate, _L_‐NAME, ascorbate + _L_‐NAME), time (six levels: baseline, exercise 1, recovery 1, exercise 2, recovery 2 at 20 and 40 min), and group (two levels: Control and T2D). Body temperatures and cardiovascular variables were analyzed using a two‐way mixed‐design analysis of variance with factors of time (six levels) and group (two levels). Absolute maximal CVC (expressed in perfusion units/mmHg) attained during sodium nitroprusside infusion was analyzed using a two‐way mixed‐design analysis of variance with factors of treatment site (four levels) and group (two levels). When a significant interaction or main effect was observed, *post hoc* multiple comparisons were conducted using the modified Bonferroni procedures [i.e., Holm procedure (Gordon and Salzman 2008)]. Student's pairwise and nonpairwise *t‐tests* were employed for between‐site and between‐group comparisons, respectively. The level of significance for all analyses was set at *P* ≤ 0.05, and all values are reported as mean ± 95% confidence interval, unless otherwise noted. All statistical tests were performed using the software package SPSS 24 for Windows (IBM, Armonk, NY).

## Results

### Participant characteristics

Age, body mass, body surface area, body fat, and absolute peak oxygen uptake were not different between groups, whereas height and peak oxygen uptake relative to body mass were lower in the T2D group (Table 1).

### Cutaneous vascular conductance

**Figure 1:**
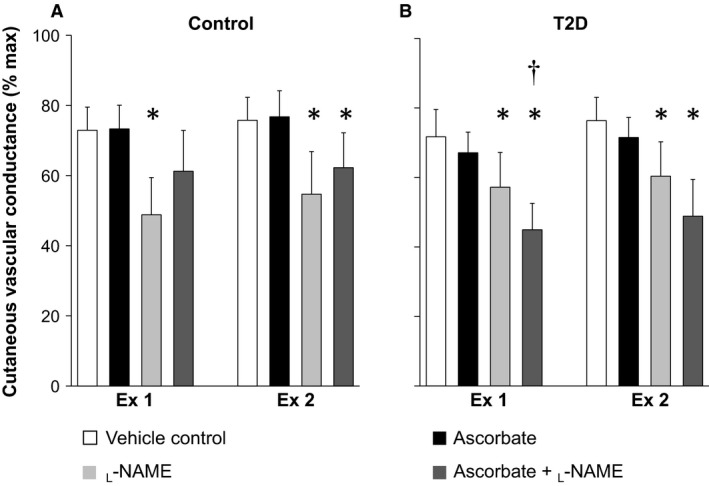
Cutaneous vascular conductance evaluated at the last 5 min of the first (Ex 1) and second (Ex 2) 30‐min exercise in older healthy adults (Control, panel A) and those with type 2 diabetes (T2D, panel B). Cutaneous vascular conductance was evaluated at four skin sites treated with either 1) lactated Ringer (vehicle control site), 2) ascorbate, an antioxidant, 3) _L_‐NAME, a nitric oxide synthase inhibitor, or 4) ascorbate + _L_‐NAME. Data are expressed as mean ± 95% confidence interval. *, versus vehicle control site (*P* ≤ 0.05). †, versus Control group (*P* ≤ 0.05).

**Figure 2:**
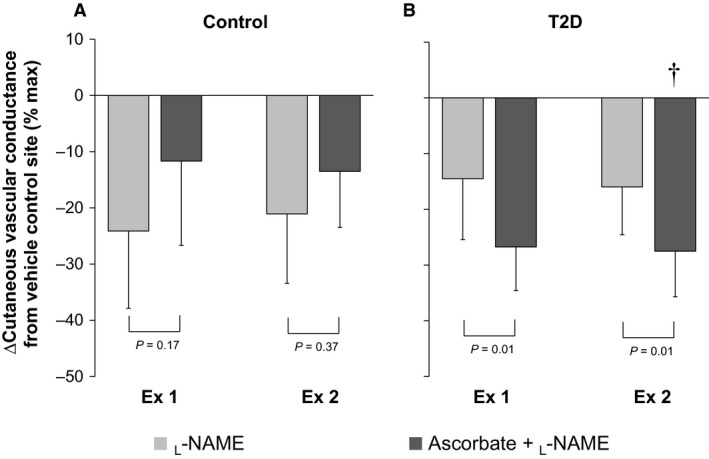
Pharmacological treatment‐induced change (∆) in cutaneous vascular conductance from lactated Ringer site (vehicle control site) in older healthy adults (Control, panel A) and those with type 2 diabetes (T2D, panel B). Cutaneous vascular conductance measured at the last 5 min of the first (Ex 1) and second (Ex 2) 30‐min exercise was used for data analysis. Pharmacological treatments employed were 1) _L_‐NAME, a nitric oxide synthase inhibitor, or 2) ascorbate (an antioxidant) + _L_‐NAME. Data are expressed as mean ± 95% confidence interval. †, versus Control group (*P* ≤ 0.05).

Main effects of treatment site, time, and group, as well as an interaction between time and group were detected for CVC (Fig. 1, Table 2, all *P* ≤ 0.05). There was an interaction between treatment site and group for ∆CVC (Fig. 2, *P* = 0.02). No main effects of treatment site and group and their interaction were measured for absolute maximal CVC (Table 2, all *P* > 0.05).

**Table 2:** Cutaneous vascular conductance measured during baseline rest (before exercise) and postexercise recovery periods as well as that evaluated during sodium nitroprusside administration (absolute maximum)

	%CVC_max_				(Perfusion units/mmHg)
	Baseline rest	Rec 1 at 20 min	Rec 2 at 20 min	Rec 2 at 40 min	Sodium nitroprusside administration
Control group					
Vehicle control	36 ± 11	44 ± 9	45 ± 10	39 ± 9	1.73 ± 0.40
Ascorbate	40 ± 8	51 ± 7	51 ± 7	51 ± 10	1.89 ± 0.43
_L_‐NAME	18 ± 5^a^	25 ± 6^a^	27 ± 9^a^	25 ± 7	1.70 ± 0.29
Ascorbate + _L_‐NAME	21 ± 7	28 ± 6^a^	25 ± 6^a^	27 ± 7	1.82 ± 0.43
T2D group					
Vehicle control	42 ± 6	57 ± 8^b^	59 ± 9^b^	53 ± 7^b^	2.02 ± 0.48
Ascorbate	42 ± 7	56 ± 9	65 ± 5^b^	58 ± 8	1.97 ± 0.52
_L_‐NAME	30 ± 10^b^	46 ± 15^b^	47 ± 11^b^	41 ± 10^b^	1.68 ± 0.32
Ascorbate + _L_‐NAME	25 ± 5^a^	37 ± 11^a^	37 ± 8^a^^,^ b	34 ± 9^a^	1.66 ± 0.40

All values are expressed as mean ± 95% confidence interval. Data were obtained by averaging values over the last 5 min at each time period. Ascorbate, an antioxidant; _L_‐NAME, a nitric oxide synthase inhibitor; Rec, recovery; T2D, type 2 diabetes; CVC, Cutaneous vascular conductance.

a versus vehicle control site (*P* ≤ 0.05).

b versus Control group (*P* ≤ 0.05).

#### Control group

In the Control group, independent infusion of ascorbate did not affect CVC relative to the vehicle control site at any time point (all *P* > 0.05, Fig. 1A and Table 2). In contrast, _L_‐NAME reduced CVC in comparison to the vehicle control site throughout the experiment (all *P* ≤ 0.05) with the exception of at 40 min into second recovery period, where CVC tended (*P* = 0.07) to be lower at the _L_‐NAME relative to vehicle control site (Fig. 1A, Table 2). Combined infusion of ascorbate and _L_‐NAME also attenuated CVC relative to the vehicle control site during the second exercise and first recovery as well as the first 20‐min of the second recovery period (all *P* ≤ 0.05, Fig. 1A, Table 2). The magnitude of reduction in CVC (∆CVC) induced by _L_‐NAME during exercise was similar with and without co‐administration of ascorbate (Fig. 2A).

#### T2D group

In the T2D group, administration of ascorbate did not affect CVC in comparison to the vehicle control site at any time point (all *P* ≤ 0.05, Fig. 1B, Table 2). _L_‐NAME attenuated CVC in relation to the vehicle control site during each exercise bout (all *P* ≤ 0.05, Fig. 1B) but not during baseline resting and each recovery periods (all *P* > 0.05, Table 2). On the other hand, _L_‐NAME in combination with ascorbate reduced CVC throughout the experiment (all *P* ≤ 0.05, Fig. 1B, Table 2). Further, when compared to the vehicle control site, the difference in CVC with _L_‐NAME administration was greater in the presence versus absence of co‐administration of ascorbate during exercise (both *P* = 0.01, Fig. 2B).

#### Between‐group comparison

During both exercise bouts, the magnitude of the reduction in CVC induced by _L_‐NAME administration alone was similar between groups (both *P* > 0.05, Fig. 2). In contrast, the reduction in CVC elicited by _L_‐NAME combined with ascorbate was greater in the T2D compared with Control group during the second exercise (*P* ≤ 0.05, Fig. 2). CVC measured at the vehicle control site did not differ between groups during baseline or each exercise, but was higher during each of the two recovery periods in the T2D relative to the Control groups (Table 2).

### Sweat rate

**Figure 3:**
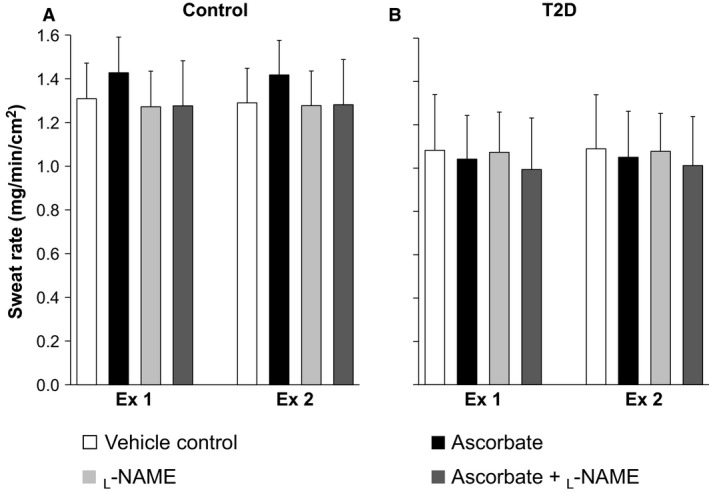
Sweat rate evaluated at the last 5 min of the first (Ex 1) and second (Ex 2) 30‐min exercise in older healthy adults (Control, panel A) and those with type 2 diabetes (T2D, panel B). Sweat rate was evaluated at four skin sites treated with either 1) lactated Ringer (vehicle control site), 2) ascorbate, an antioxidant, 3) _L_‐NAME, a nitric oxide synthase inhibitor, or 4) ascorbate + _L_‐NAME. Data are expressed as mean ± 95% confidence interval. Values did not differ between sites and groups (all *P* > 0.07).

A main effect of time and an interaction between time and group were detected for sweat rate (Fig. 3, Table 3, all *P* ≤ 0.05). However, there were no between‐site or between‐group differences in sweat rate throughout the experiment (all *P* > 0.05, Fig. 3 and Table 3).

**Table 3:** Sweat rate measured during baseline rest (before exercise) and postexercise recovery periods

	(mg/min/cm^2^)			
	Baseline rest	Rec 1 at 20 min	Rec 2 at 20 min	Rec 2 at 40 min
Control group				
Vehicle control	0.25 ± 0.05	0.62 ± 0.08	0.70 ± 0.10	0.49 ± 0.07
Ascorbate	0.26 ± 0.06	0.72 ± 0.08	0.79 ± 0.10	0.60 ± 0.07
_L_‐NAME	0.24 ± 0.05	0.69 ± 0.08	0.72 ± 0.10	0.53 ± 0.07
Ascorbate + _L_‐NAME	0.26 ± 0.05	0.65 ± 0.11	0.72 ± 0.13	0.52 ± 0.09
T2D group				
Vehicle control	0.35 ± 0.07	0.81 ± 0.23	0.94 ± 0.27	0.71 ± 0.18
Ascorbate	0.34 ± 0.06	0.71 ± 0.16	0.81 ± 0.17	0.64 ± 0.13
_L_‐NAME	0.35 ± 0.07	0.77 ± 0.16	0.81 ± 0.15	0.67 ± 0.13
Ascorbate + _L_‐NAME	0.34 ± 0.07	0.68 ± 0.20	0.77 ± 0.21	0.63 ± 0.19

All values are expressed as mean ± 95% confidence interval. Data were obtained by averaging values over the last 5 min at each time period. Ascorbate, an antioxidant; _L_‐NAME, a nitric oxide synthase inhibitor; Rec, recovery; T2D, type 2 diabetes. There were no between‐site and between‐group differences (all *P* > 0.07).

### Cardiovascular and thermal responses

A main effect of time was found for heart rate, whereas a main effect of time and an interaction between time and group were measured on mean skin temperature and mean arterial pressure (Table 4, all *P* ≤ 0.05). Main effects of time and group and an interaction between time and group were detected for body core temperature (Table 4, all *P* ≤ 0.05). Heart rate, mean arterial pressure, and mean skin temperature did not differ between groups throughout the protocol (all *P* > 0.05) with the exception of lower mean skin temperature during baseline rest in the T2D group (*P* ≤ 0.05, Table 4). Body core temperature was higher in the T2D group as compared to the Control group during the second exercise and both recovery periods (all *P* ≤ 0.05, Table 4).

**Table 4:** Cardiovascular and body temperature responses measured during baseline rest (before exercise) and the two exercise and recovery periods

	Baseline rest	Ex 1 at 30 min	Rec 1 at 20 min	Ex 2 at 30 min	Rec 2	
					at 20 min	at 40 min
Heart rate (bpm)						
Control group	71 ± 5	121 ± 10^a^	85 ± 9^a^	128 ± 12^a^^,^ b	90 ± 11^a^^,^ b	86 ± 9^a^
T2D group	77 ± 8	128 ± 11^a^	100 ± 13^a^	136 ± 11^a^^,^ b	106 ± 13^a^^,^ b	98 ± 11^a^
Mean arterial pressure (mmHg)						
Control group	99 ± 4	107 ± 5^a^	95 ± 5	105 ± 6	93 ± 4^a^	91 ± 4^a^^,^ b
T2D group	97 ± 5	112 ± 3^a^	96 ± 4	109 ± 5^a^	92 ± 5	89 ± 5^a^^,^ b
Mean skin temperature (°C)						
Control group	34.94 ± 0.30	35.37 ± 0.31^a^	34.86 ± 0.33	35.35 ± 0.49	34.96 ± 0.35	34.43 ± 0.35^a^^,^ b
T2D group	34.38 ± 0.24^c^	35.50 ± 0.37^a^	35.30 ± 0.27^a^	35.65 ± 0.43^a^	35.42 ± 0.39^a^	34.92 ± 0.42^a^^,^ b
Body core temperature (°C)						
Control group	37.01 ± 0.16	37.81 ± 0.18^a^	37.37 ± 0.10^a^	37.99 ± 0.24^a^	37.45 ± 0.14^a^	37.35 ± 0.13^a^
T2D group	37.07 ± 0.20	37.98 ± 0.19^a^	37.81 ± 0.21^a^^,^ c	38.34 ± 0.23^a^^,^ b ^,^ c	38.18 ± 0.31^a^^,^ b ^,^ c	37.87 ± 0.23^a^^,^ c

Values are mean ± 95% confidence interval. Data were obtained by averaging values over the last 5 min at each time period.

Ex, exercise; Rec, recovery; T2D, type 2 diabetes. bpm, beats per minute.

a versus baseline rest (*P* ≤ 0.05).

b Ex 1 versus 2 or Rec 1 versus 2 (*P* ≤ 0.05).

c versus Control group (*P* ≤ 0.05).

### Blood glucose

Blood glucose in the T2D group at baseline rest was 8.8 ± 1.2 mmol/L, which decreased to 7.3 ± 0.7 mmol/L at the end of second recovery (*P* = 0.05).

### Body weight loss

A similar reduction in body weight following exercise in the heat was measured in the Control and T2D groups (1.4 ± 0.1 vs. 1.6 ± 0.3 kg, *P* = 0.27).

## Discussion

We are the first to examine the effect of intradermal ascorbate administration on the regulation of cutaneous vasodilation and sweating in healthy older adults and those with type 2 diabetes exercising in the heat. Our results suggest that during exercise in the heat, ascorbate does not modulate the heat loss responses of cutaneous vasodilation and sweating in both healthy older adults and those with type 2 diabetes. However, we show that NOS is an important contributor to the regulation of cutaneous vasodilation in both groups.

### Cutaneous vascular response

Our results demonstrated no effect of ascorbate on CVC during exercise in the heat in healthy older adults (Fig. 1A) and this response remained intact during baseline rest as well as post‐exercise recovery (Table 2). This is in contrast to a previous report by Holowatz et al. (2006) in which ascorbate augmented cutaneous vasodilation during passive heating at rest using a water‐perfusion suit. The reason underlying this discrepancy is unknown, but the influence of ascorbate on cutaneous vasodilation during heat stress may differ depending on the level of mean skin temperature. Mean skin temperature in the present study was ~35°C (Table 4). In contrast, while mean skin temperature was not reported by Holowatz et al. (2006), the temperature measured in their study would likely have been higher (e.g., ~39°C). This is supported by the observations by Brunt et al. (2013) using a similar heating procedure with a water‐perfusion suit. Higher skin temperature may induce greater increase in oxidative stress, as a previous report demonstrated that oxidative marker (malondialdehyde) was greater during exercise performed in moderate (32°C) versus cool (12°C) ambient temperature conditions (Sureda et al. 2015). Hence, the level of oxidative stress in the present study might not have been high enough to affect cutaneous vascular response.

We show for the first time that ascorbate had no effect on cutaneous vasodilation during a passive or exercise‐induced heat stress in older adults with well controlled type 2 diabetes (Fig. 1B, Table 2). Although ascorbate may be effective in improving vasodilation response in forearm conduit artery in individuals with type 2 diabetes (Montero et al. 2014), its influence on markers of oxidative stress (Rytter et al. 2010) and diabetic complications (Rahimi‐Madiseh et al. 2016) are relatively minor; a response comparable to that observed on skin vessels in the present study. It should be noted that we employed individuals with well‐controlled type 2 diabetes without the presence of peripheral neuropathy or other diabetic complications. Sweating function may be further impaired in individuals with poor glycemic control and/or the presence of peripheral neuropathy (Luo et al. 2012; Kenny et al. 2016b). Thus, the potential exists that the therapeutic benefits of ascorbate in enhancing heat dissipation may be most evident in heat‐vulnerable individuals. However, this possibility requires future scrutiny.

The present study demonstrated that NOS contributes to the regulation of cutaneous vasodilation during exercise in the heat in relatively healthy older adults (Fig. 1A), which is in accordance with previous reports using passive heating at rest (Holowatz et al. 2003; Stanhewicz et al. 2012) and exercise in the heat (Fujii et al. 2015b, 2016b). Our results also demonstrate that NOS contributes to cutaneous vasodilation in individuals with T2D during exercise in the heat (Fig. 1B). Furthermore, the magnitude of the contribution of NOS as evaluated by the _L_‐NAME‐induced reduction in CVC from the vehicle control site did not differ between groups during exercise in the heat (Fig. 2). This is consistent with a previous report assessing the response during passive heating at rest (Sokolnicki et al. 2009). Therefore, our results in combinations with the findings of others (Sokolnicki et al. 2009) suggest that type 2 diabetes does not blunt NOS‐dependent cutaneous vasodilation irrespective of the nature of the heat stress (i.e., a passive or exercise‐induced heat stress).

Interestingly, we observed that the influence of the combined administration of ascorbate and _L_‐NAME on cutaneous vasodilation during exercise differed between the Control and T2D groups (Fig. 2). Specifically, in the Control group, the reduction in CVC induced by the combined administration of ascorbate and _L_‐NAME was similar to the response measured with the administration of _L_‐NAME only (Fig. 2A). In contrast, we showed that the reduction in CVC was greater than that achieved with _L_‐NAME alone in the T2D group (Fig. 2B). Based on these results, it appears that in the absence of NO production, ascorbate seems to further attenuate cutaneous vasodilation in individuals with type 2 diabetes. More work is required to assess the mechanisms underpinning this response.

We found that CVC measured at the vehicle control site was higher during the recovery periods in the T2D as compared with the Control group (Table 2). It is unclear why the T2D group exhibited a higher CVC response during the postexercise recovery period only, but a higher body core temperature and therefore greater thermal drive may be involved (Table 4). In contrast to our findings, previous studies have shown that type 2 diabetes attenuates cutaneous vasodilation during passive heating at rest (Wick et al. 2006; Sokolnicki et al. 2009). However, these previous studies employed a different heat stress protocol (whole‐body heating with a water‐perfused suit) that makes a direct comparison of responses difficult.

### Sweating response

We found that ascorbate did not influence sweat rate relative to the vehicle control site during exercise (Fig. 3) as well as during resting and postexercise recovery (Table 3) in the heat in either group. Hence, ascorbate does not appear to enhance sweating in healthy older adults and those with type 2 diabetes under the conditions examined. On the other hand, in keeping with our previous studies (Stapleton et al. 2014; Fujii et al. 2015b, 2016b), we confirmed no role for NOS in the regulation of sweating during exercise in the heat in healthy older adults (Fig. 3), which is in contrast to findings in younger adults (Welch et al. 2009; Stapleton et al. 2014; Fujii et al. 2016b). Since no contribution of NOS to sweating during exercise in the heat was also observed in the T2D group (Fig. 3), the age‐related reduction in NOS‐dependent sweating is present irrespective of the presence of type 2 diabetes. While the mechanisms underpinning these findings are currently unclear, increased oxidative stress associated with aging might not be involved given that ascorbate perfusion did not influence the sweating response when administered alone or in combination with _L_‐NAME. Further research is warranted to delineate the mechanisms mediating the age‐related attenuation in NOS‐dependent sweating during exercise in the heat.

As with cutaneous vasodilation, our results demonstrate that type 2 diabetes does not modulate local forearm sweat rate during exercise in the heat (Fig. 3), which is consistent with our previous work (Kenny et al. 2013). Noteworthy, the same study showed that whole‐body sweating during exercise is indeed attenuated in individuals with T2D (Kenny et al. 2013). Therefore, it is likely that the influence of type 2 diabetes on sweating during exercise in the heat is site‐specific. Attenuations in whole‐body sweating and thus heat loss is indirectly supported by the fact that we observed a greater increase in body core temperature in the T2D versus Control group after the first exercise (Table 4). Further studies are required to evaluate regional responses associated with diabetes‐related modulation of the sweating response.

### Considerations

We did not directly assess levels of ascorbate/vitamin C and oxidative stress in the Control and T2D groups. It is therefore possible that our findings are simply a product of including participants in both groups with relatively similar levels of systemic inflammation/oxidative stress. By design, we examined the influence of local ascorbate infusion in individuals with well‐controlled type 2 diabetes. It is possible however, that relative to their healthy counterparts, differences in levels of systemic inflammation/oxidative stress may be more pronounced in individuals with type 2 diabetes who have poor glycemic control and or who are affected by diabetes‐related complications. Factors such as medication use may also play an important role in modulating this response. However, given our T2D participants were on several different medications, our results do not provide information pertaining to which medication may have influenced the observed findings.

### Perspectives and significance

Older individuals are at greater risk of heat‐related injuries (Worfolk 2000; Kenney et al. 2014; Kenny et al. 2016b), and type 2 diabetes can exacerbate this risk (Semenza et al. 1999; Kenny et al. 2016b). Hence the development of interventions to augment heat dissipation and circumvent potentially dangerous increases in body core temperature is of the utmost importance. Based on the results obtained in this study, we show that ascorbate does not improve the heat loss responses through cutaneous vasodilation and sweating in healthy older adults and those with type 2 diabetes during exercise as well as resting before and after exercise in the heat. Therefore, oral supplementation of ascorbate may not be an effective therapeutic strategy to reverse age‐ and diabetes‐related impairments in heat loss in these at‐risk populations.

## Conclusion

We show that intradermal administration of ascorbate does not affect cutaneous vasodilation and sweating during exercise in the heat in both healthy older adults and those with type 2 diabetes. We also show that in both groups, NOS contributes to the regulation of cutaneous perfusion during exercise in the heat.

## Conflict of Interest

None.

## References

[phy213238-bib-0001] Anderson, C. , T. Andersson , and K. Wardell . 1994 Changes in skin circulation after insertion of a microdialysis probe visualized by laser Doppler perfusion imaging. J. Invest. Dermatol. 102:807–811. 8176267 10.1111/1523-1747.ep12378630

[phy213238-bib-0002] Brunt, V. E. , N. Fujii , and C. T. Minson . 2013 No independent, but an interactive, role of calcium‐activated potassium channels in human cutaneous active vasodilation. J. Appl. Physiol. 115:1290–1296. 23970531 10.1152/japplphysiol.00358.2013 PMC3841827

[phy213238-bib-0003] Casoinic, F. , D. Sampelean , A. D. Buzoianu , N. Hancu , and D. Baston . 2016 Serum Levels of Oxidative Stress Markers in Patients with Type 2 Diabetes Mellitus and Non‐alcoholic Steatohepatitis. Rom. J. Intern. Med. 54:228–236. 28002036 10.1515/rjim-2016-0035

[phy213238-bib-0004] DuBois, D. , and E. F. DuBois . 1916 A formula to estimate the approximate surface area if height and weight be known. Arch. Intern. Med. 17:863–871.

[phy213238-bib-0005] Durrant, J. R. , D. R. Seals , M. L. Connell , M. J. Russell , B. R. Lawson , B. J. Folian , et al. 2009 Voluntary wheel running restores endothelial function in conduit arteries of old mice: direct evidence for reduced oxidative stress, increased superoxide dismutase activity and down‐regulation of NADPH oxidase. J. Physiol. 587:3271–3285. 19417091 10.1113/jphysiol.2009.169771 PMC2727036

[phy213238-bib-0006] Fealey, R. D. , P. A. Low , and J. E. Thomas . 1989 Thermoregulatory sweating abnormalities in diabetes mellitus. Mayo Clin. Proc. 64:617–628. 2747292 10.1016/s0025-6196(12)65338-5

[phy213238-bib-0007] Folli, F. , D. Corradi , P. Fanti , A. Davalli , A. Paez , A. Giaccari , et al. 2011 The role of oxidative stress in the pathogenesis of type 2 diabetes mellitus micro‐ and macrovascular complications: avenues for a mechanistic‐based therapeutic approach. Curr. Diabetes Rev. 7:313–324. 21838680 10.2174/157339911797415585

[phy213238-bib-0008] Fujii, N. , V. E. Brunt , and C. T. Minson . 2014a Tempol improves cutaneous thermal hyperemia through increasing nitric oxide bioavailability in young smokers. Am. J. Physiol. Heart Circ. Physiol. 306:H1507–H1511. 24682395 10.1152/ajpheart.00886.2013 PMC4042197

[phy213238-bib-0009] Fujii, N. , R. McGinn , J. M. Stapleton , G. Paull , R. D. Meade , and G. P. Kenny . 2014b Evidence for cyclooxygenase‐dependent sweating in young males during intermittent exercise in the heat. J. Physiol. 592:5327–5339. 25326453 10.1113/jphysiol.2014.280651 PMC4262342

[phy213238-bib-0010] Fujii, N. , R. D. Meade , G. Paull , R. McGinn , I. Foudil‐Bey , P. Akbari , et al. 2015a Can intradermal administration of angiotensin II influence human heat loss responses during whole‐body heat stress?. J. Appl. Physiol. (1985) 118:1145–1153. 25767030 10.1152/japplphysiol.00025.2015 PMC4421789

[phy213238-bib-0011] Fujii, N. , G. Paull , R. D. Meade , R. McGinn , J. M. Stapleton , P. Akbari , et al. 2015b Do nitric oxide synthase and cyclooxygenase contribute to the heat loss responses in older males exercising in the heat? J. Physiol. 593:3169–3180. 25820454 10.1113/JP270330 PMC4532535

[phy213238-bib-0012] Fujii, N. , S. Dervis , R. J. Sigal , and G. P. Kenny . 2016a Type 1 diabetes modulates cyclooxygenase‐ and nitric oxide‐dependent mechanisms governing sweating but not cutaneous vasodilation during exercise in the heat. Am. J. Physiol. Regul. Integr. Comp. Physiol. 311:R1076–R1084. 27733388 10.1152/ajpregu.00376.2016

[phy213238-bib-0013] Fujii, N. , R. D. Meade , L. M. Alexander , P. Akbari , I. Foudil‐Bey , J. C. Louie , et al. 2016b iNOS‐dependent sweating and eNOS‐dependent cutaneous vasodilation are evident in younger adults, but are diminished in older adults exercising in the heat. J. Appl. Physiol. (1985) 120:318–327. 26586908 10.1152/japplphysiol.00714.2015 PMC4740499

[phy213238-bib-0014] Gagnon, D. , O. Jay , and G. P. Kenny . 2013 The evaporative requirement for heat balance determines whole‐body sweat rate during exercise under conditions permitting full evaporation. J. Physiol. 591:2925–2935. 23459754 10.1113/jphysiol.2012.248823 PMC3690695

[phy213238-bib-0015] Gordon, A. Y. , and P. Salzman . 2008 Optimality of the Holm procedure among general step‐down multiple testing procedures. Stat Probab Lett 78:1878–1884. 19759804 10.1016/j.spl.2008.01.055 PMC2583789

[phy213238-bib-0016] Hardy, J. D. , and E. F. Dubois . 1938 The technic of measuring radiation and convection. J. Nutr. 15:461–475.

[phy213238-bib-0017] Holowatz, L. A. , and W. L. Kenney . 2007 Local ascorbate administration augments NO‐ and non‐NO‐dependent reflex cutaneous vasodilation in hypertensive humans. Am. J. Physiol. Heart Circ. Physiol. 293:H1090–H1096. 17483240 10.1152/ajpheart.00295.2007

[phy213238-bib-0018] Holowatz, L. A. , B. L. Houghton , B. J. Wong , B. W. Wilkins , A. W. Harding , W. L. Kenney , et al. 2003 Nitric oxide and attenuated reflex cutaneous vasodilation in aged skin. Am. J. Physiol. Heart Circ. Physiol. 284:H1662–H1667. 12505876 10.1152/ajpheart.00871.2002

[phy213238-bib-0019] Holowatz, L. A. , C. S. Thompson , and W. L. Kenney . 2006 Acute ascorbate supplementation alone or combined with arginase inhibition augments reflex cutaneous vasodilation in aged human skin. Am. J. Physiol. Heart Circ. Physiol. 291:H2965–H2970. 16905599 10.1152/ajpheart.00648.2006

[phy213238-bib-0020] Kellogg, D. L. Jr , C. G. Crandall , Y. Liu , N. Charkoudian , and J. M. Johnson . 1998 Nitric oxide and cutaneous active vasodilation during heat stress in humans. J. Appl. Physiol. 85:824–829. 9729553 10.1152/jappl.1998.85.3.824

[phy213238-bib-0021] Kenney, W. L. , D. H. Craighead , and L. M. Alexander . 2014 Heat Waves, Aging and Human Cardiovascular Health. Med. Sci. Sports Exerc. 46:1891–1899. 24598696 10.1249/MSS.0000000000000325 PMC4155032

[phy213238-bib-0022] Kenny, G. P. , and O. Jay . 2013 Thermometry, Calorimetry, and Mean Body Temperature during Heat Stress. Compr. Physiol. 3:1689–1719. 24265242 10.1002/cphy.c130011

[phy213238-bib-0023] Kenny, G. P. , J. M. Stapleton , J. E. Yardley , P. Boulay , and R. J. Sigal . 2013 Older Adults with Type 2 Diabetes Store More Heat during Exercise. Med. Sci. Sports Exerc. 45:1906–1914. 23542894 10.1249/MSS.0b013e3182940836

[phy213238-bib-0024] Kenny, G. P. , A. D. Flouris , S. Dervis , B. J. Friesen , R. J. Sigal , J. Malcolm , et al. 2015 Older adults experience greater levels of thermal and cardiovascular strain during extreme heat exposures. Med. Sci. Sports Exerc. 47(5s):497.

[phy213238-bib-0025] Kenny, G. P. , M. P. Poirier , G. S. Metsios , P. Boulay , S. Dervis , B. J. Friesen , et al. 2016a Hyperthermia and cardiovascular strain during an extreme heat exposure in young versus older adults. Temperature (Austin) 3:1–10. 28349096 10.1080/23328940.2016.1230171 PMC5356213

[phy213238-bib-0026] Kenny, G. P. , R. J. Sigal , and R. McGinn . 2016b Body temperature regulation in diabetes. Temperature (Austin) 3:119–145. 27227101 10.1080/23328940.2015.1131506 PMC4861190

[phy213238-bib-0027] Louie, J. C. , N. Fujii , R. D. Meade , and G. P. Kenny . 2016 The interactive contributions of Na/K ‐ATPase and nitric oxide synthase to sweating and cutaneous vasodilation during exercise in the heat. J. Physiol. 594:3453–3462. 26852741 10.1113/JP271990 PMC4908024

[phy213238-bib-0028] Luo, K. R. , C. C. Chao , P. C. Hsieh , J. H. Lue , and S. T. Hsieh . 2012 Effect of glycemic control on sudomotor denervation in type 2 diabetes. Diabetes Care 35:612–616. 22301122 10.2337/dc11-1607 PMC3322711

[phy213238-bib-0029] McNamara, T. C. , J. T. Keen , G. H. Simmons , L. M. Alexander , and B. J. Wong . 2014 Endothelial nitric oxide synthase mediates the nitric oxide component of reflex cutaneous vasodilatation during dynamic exercise in humans. J. Physiol. 592:5317–5326. 25260636 10.1113/jphysiol.2014.272898 PMC4262341

[phy213238-bib-0030] Meade, R. D. , N. Fujii , L. M. Alexander , G. Paull , J. C. Louie , A. D. Flouris , et al. 2015 Local infusion of ascorbate augments NO‐dependent cutaneous vasodilatation during intense exercise in the heat. J. Physiol. 593:4055–4065. 26110415 10.1113/JP270787 PMC4575586

[phy213238-bib-0031] Meade, R. D. , J. C. Louie , M. P. Poirier , R. McGinn , N. Fujii , and G. P. Kenny . 2016 Exploring the mechanisms underpinning sweating: the development of a specialized ventilated capsule for use with intradermal microdialysis. Physiol. Rep. 4:e12738. 27033452 10.14814/phy2.12738 PMC4814883

[phy213238-bib-0032] Medow, M. S. , N. Bamji , D. Clarke , A. J. Ocon , and J. M. Stewart . 2011 Reactive Oxygen Species (ROS) from NADPH and Xanthine Oxidase Modulate the Cutaneous Local Heating Response in Healthy Humans. J. Appl. Physiol. 111:20–26. 21436462 10.1152/japplphysiol.01448.2010 PMC3137526

[phy213238-bib-0033] Montero, D. , G. Walther , C. D. Stehouwer , A. J. Houben , J. A. Beckman , and A. Vinet . 2014 Effect of antioxidant vitamin supplementation on endothelial function in type 2 diabetes mellitus: a systematic review and meta‐analysis of randomized controlled trials. Obes. Rev. 15:107–116. 10.1111/obr.12114 24118784

[phy213238-bib-0034] Nishi, Y . 1981 Measurement of thermal balance in man Pp. 29–39 in CJ Cena K Bioengineering, Thermal Physiology and Comfort, ed., Elsevier, New York.

[phy213238-bib-0035] Petrofsky, J. S. , S. Lee , C. Patterson , M. Cole , and B. Stewart . 2005 Sweat production during global heating and during isometric exercise in people with diabetes. Med. Sci. Monit. 11:CR515–CR521. 16258395

[phy213238-bib-0036] Rahimi‐Madiseh, M. , A. Malekpour‐Tehrani , M. Bahmani , and M. Rafieian‐Kopaei . 2016 The research and development on the antioxidants in prevention of diabetic complications. Asian Pac. J. Trop. Med. 9:825–831. 27633293 10.1016/j.apjtm.2016.07.001

[phy213238-bib-0037] Restaino, R. M. , S. H. Deo , A. R. Parrish , P. J. Fadel , and J. Padilla . 2016 Increased monocyte‐derived reactive oxygen species in type 2 diabetes: role of endoplasmic reticulum stress. Exp. Physiol. 102:139–153. 10.1113/EP085794 PMC5600886 27859785

[phy213238-bib-0038] Rytter, E. , B. Vessby , R. Asgard , C. Ersson , S. Moussavian , A. Sjodin , et al. 2010 Supplementation with a combination of antioxidants does not affect glycaemic control, oxidative stress or inflammation in type 2 diabetes subjects. Free Radic. Res. 44:1445–1453. 20942575 10.3109/10715762.2010.515219

[phy213238-bib-0039] Semenza, J. C. , J. E. McCullough , W. D. Flanders , M. A. McGeehin , and J. R. Lumpkin . 1999 Excess hospital admissions during the July 1995 heat wave in Chicago. Am. J. Prev. Med. 16:269–277. 10493281 10.1016/s0749-3797(99)00025-2

[phy213238-bib-0040] Shastry, S. , C. T. Minson , S. A. Wilson , N. M. Dietz , and M. J. Joyner . 2000 Effects of atropine and L‐NAME on cutaneous blood flow during body heating in humans. J. Appl. Physiol. 88:467–472. 10658012 10.1152/jappl.2000.88.2.467

[phy213238-bib-0041] Siri, W. E. 1956 The gross composition of the body. Adv. Biol. Med. Phys. 4:239–280. 13354513 10.1016/b978-1-4832-3110-5.50011-x

[phy213238-bib-0042] Sokolnicki, L. A. , N. A. Strom , S. K. Roberts , S. A. Kingsley‐Berg , A. Basu , and N. Charkoudian . 2009 Skin blood flow and nitric oxide during body heating in type 2 diabetes mellitus. J. Appl. Physiol. (1985) 106:566–570. 19056994 10.1152/japplphysiol.91289.2008 PMC2644253

[phy213238-bib-0043] Stanhewicz, A. E. , R. S. Bruning , C. J. Smith , W. L. Kenney , and L. A. Holowatz . 2012 Local tetrahydrobiopterin administration augments reflex cutaneous vasodilation through nitric oxide‐dependent mechanisms in aged human skin. J. Appl. Physiol. (1985) 112:791–797. 22162527 10.1152/japplphysiol.01257.2011 PMC3311663

[phy213238-bib-0044] Stanhewicz, A. E. , L. M. Alexander , and W. L. Kenney . 2013 Oral sapropterin acutely augments reflex vasodilation in aged human skin through nitric oxide‐dependent mechanisms. J. Appl. Physiol. 115:972–978. 23743404 10.1152/japplphysiol.00481.2013 PMC3798819

[phy213238-bib-0045] Stapleton, J. M. , N. Fujii , M. Carter , and G. P. Kenny . 2014 Diminished nitric oxide‐dependent sweating in older males during intermittent exercise in the heat. Exp. Physiol. 99:921–932. 24706193 10.1113/expphysiol.2013.077644

[phy213238-bib-0046] Stewart, J. M. , I. Taneja , N. Raghunath , D. Clarke , and M. S. Medow . 2008 Intradermal angiotensin II administration attenuates the local cutaneous vasodilator heating response. Am. J. Physiol. Heart Circ. Physiol. 295:H327–H334. 18469148 10.1152/ajpheart.00126.2008 PMC2494761

[phy213238-bib-0047] Sureda, A. , A. Mestre‐Alfaro , M. Banquells , J. Riera , F. Drobnic , J. Camps , et al. 2015 Exercise in a hot environment influences plasma anti‐inflammatory and antioxidant status in well‐trained athletes. J. Therm. Biol 47:91–98. 25526659 10.1016/j.jtherbio.2014.11.011

[phy213238-bib-0048] Welch, G. , K. M. Foote , C. Hansen , and G. W. Mack . 2009 Nonselective NOS inhibition blunts the sweat response to exercise in a warm environment. J. Appl. Physiol. 106:796–803. 19131481 10.1152/japplphysiol.90809.2008 PMC2660248

[phy213238-bib-0049] Wick, D. E. , S. K. Roberts , A. Basu , P. Sandroni , R. D. Fealey , D. Sletten , et al. 2006 Delayed threshold for active cutaneous vasodilation in patients with Type 2 diabetes mellitus. J. Appl. Physiol. 100:637–641. 16210432 10.1152/japplphysiol.00943.2005

[phy213238-bib-0050] Worfolk, J. B. 2000 Heat waves: their impact on the health of elders. Geriatr. Nurs. 21:70–77. 10769330 10.1067/mgn.2000.107131

[phy213238-bib-0051] Yamazaki, F . 2010 Local ascorbate administration inhibits the adrenergic vasoconstrictor response to local cooling in the human skin. J. Appl. Physiol. (1985) 108:328–333. 20007855 10.1152/japplphysiol.00814.2009

